# A Flexible Electrochemiluminescence Sensor Equipped With Vertically Ordered Mesoporous Silica Nanochannel Film for Sensitive Detection of Clindamycin

**DOI:** 10.3389/fchem.2022.872582

**Published:** 2022-04-06

**Authors:** Xinjie Wei, Xuan Luo, Shuai Xu, Fengna Xi, Tingting Zhao

**Affiliations:** ^1^ Guangxi Medical University Cancer Hospital, Guangxi Medical University, Nanning, China; ^2^ Department of Chemistry, Key Laboratory of Surface and Interface Science of Polymer Materials of Zhejiang Province, Zhejiang Sci-Tech University, Hangzhou, China

**Keywords:** electrochemiluminescence sensor, flexible electrode, vertically ordered mesoporous silica nanochannel film, sensitive detection, clindamycin

## Abstract

Fast, convenient, and highly sensitive detection of antibiotic is essential to avoid its overuse and the possible harm. Owing to enrichment effect and antifouling ability of ultrasmall nanochannels, the vertically ordered mesoporous silica nanochannel film (VMSF) has great potential in the development of the facile electrochemiluminescence (ECL) sensor for direct and sensitive analysis of antibiotics in complex samples. In this study, we demonstrated a flexible ECL sensor based on a cost-effective electrode covered with a VMSF for sensitive detection of clindamycin. Polyethylene terephthalate coated with indium tin oxide (PET-ITO) is applied as a flexible electrode to grow VMSF using the electrochemically assisted self-assembly (EASA) method. The negatively charged VMSF nanochannels exhibit significant enrichment toward the commonly used cationic ECL luminophores, tris(2,2-bipyridyl) dichlororuthenium (II) (Ru (bpy)_3_^2+^). Using the enhanced ECL of Ru (bpy)_3_^2+^ by clindamycin, the developed VMSF/PET-ITO sensor can sensitively detect clindamycin. The responses were linear in the concentration range of 10 nM–25 μM and in the concentration range of 25–70 μM. Owing to the nanoscale thickness of the VMSF and the high coupling stability with the electrode substrate, the developed flexible VMSF/PET-ITO sensor exhibits high signal stability during the continuous bending process. Considering high antifouling characteristic of the VMSF, direct analysis of clindamycin in a real biological sample, human serum, is realized.

## Introduction

Antibiotics, drugs that can inhibit and kill bacteria, have been widely used in medical and healthcare, livestock and poultry breeding, agricultural production, and other industries. However, the excessive and irrational use of antibiotics is now widespread. Antibiotic residues can cause serious harm to human health including carcinogenic, teratogenic, and mutagenic effects (Wang et al., 2008; Wong et al., 2016; Hadi and Honarmand, 2017; Paul et al., 2017). In addition, prolonged exposure to such drugs can lead to severe antibiotic-resistant infections. For instance, clindamycin is a lincosamide antibiotic, which is widely used for the treatment of bacterial infections. The side effects of clindamycin include dry skin, nausea, vomiting, and constipation (Liu et al., 2010; Habib et al., 2011; Girondi et al., 2018; Mohammad Beigi et al., 2020; Liang et al., 2021). Researchers are also highly concerned about the bacterial resistance of clindamycin due to its overuse. Therefore, rapid, low-cost, and highly sensitive detection of antibiotics is crucial to standardize the use of antibiotics and avoid harm.

Until now, the developed technologies for the detection of antibiotics include capillary electrophoresis (CE), high-performance liquid chromatography (HLPC), ultrahigh-performance liquid chromatography–electrospray tandem mass spectrometry (UPLC-ESI-MS/MS), and electrochemical sensors (Liu et al., 2010; Habib et al., 2011; Hadi and Honarmand, 2017; Girondi et al., 2018; Mohammad Beigi et al., 2020; Liang et al., 2021). However, these methods usually suffer time-consuming detection, expensive instruments, complicated operation, or low sensitivity. Electrochemiluminescence (ECL), where chemiluminescence (CL) is triggered and controlled by electrochemical methods, elegantly combines the electrochemical and optical methods (Chen et al., 2011; Li et al., 2017; Zhai et al., 2017; Ma et al., 2019). Compared with photoluminescence with photo-excitation, ECL has advantages of near-zero background, high detection sensitivity, simplified optical instrument, and good temporal/spatial resolution (Wang et al., 2020; Zhang et al., 2020; Wang et al., 2021a; Huang et al., 2021; Ma et al., 2021; Yang et al., 2021; Zhu et al., 2021). Thus, ECL has emerged as a powerful tool in clinical diagnosis, drug analysis, immunoassay, environmental analysis, etc. In addition to constructing an ECL sensor on conventional electrochemical electrodes, development of a flexible, cost-efficient, and disposable ECL sensor with high sensitivity and its application in sensitive detection of antibiotic is of great significance.

The modification of the supporting electrode can significantly affect the performance of ECL sensors. As functional nanomaterials in molecular sieves, nanofluids, sensing and energy conversion, solid-state nanofilms (SSN) have attracted great attention owing to advantages of tunable nanopores, high specific surface area, and intelligent control of molecular transport (Zhou et al., 2019a; Zhou et al., 2019b; Zhou et al., 2020a; Ding et al., 2020; Li et al., 2020). Among the developed SSN, vertically ordered mesoporous silica nanochannel film (VMSF) exhibits high chemical and thermal stability, ordered and vertically aligned nanochannels (nanopores), uniform pore size distribution, high pore density, and ease of modification (Nasir et al., 2018; Jiokeng et al., 2019; Ullah et al., 2021; Walcarius, 2021). The ultrasmall (usually 2–3 nm) and high-density (up to 4∼8 × 10^12^/cm^2^) nanochannel arrays make it possible to significantly improve the sensor performance as electrode modification materials (Mi et al., 2020; Li et al., 2021a; Asadpour et al., 2021; Li et al., 2021b; Xiao et al., 2021). On the one hand, a large number of silanol groups (Si-OH) with low pKa (∼2) endow the VMSF with a negatively charged surface, which can accelerate the transfer of positively charged molecules to the electrode surface, leading to a significant enrichment effect. On the other hand, ultrasmall nanochannels of the VMSF can exclude large-sized substances, which can effectively suppress the interference of coexisting substances in complex samples, providing high antifouling ability and good signal stability (Yan et al., 2020a; Yan et al., 2020b; Lin et al., 2020; Liu et al., 2020; Ma et al., 2020; Yan et al., 2021a; Wang et al., 2021b; Yan et al., 2021b; Qiu et al., 2021; Yanyan et al., 2021). Therefore, the VMSF has great potential in the development of facile ECL sensors for direct and sensitive analysis of antibiotics in complex samples.

**FIGURE 1:**
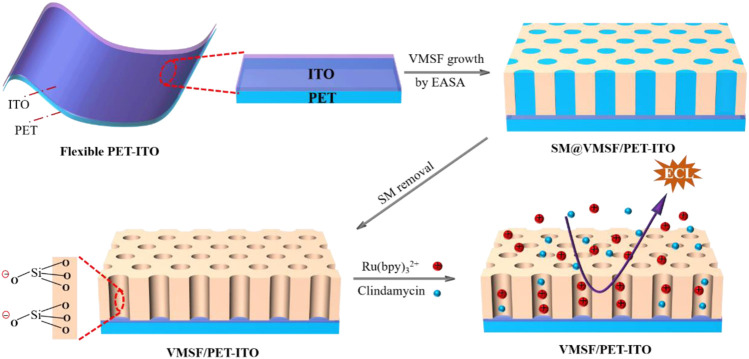
Illustration for the preparation of VMSF/PET-ITO using the EASA method and the enrichment of Ru (bpy)_3_^2+^ on negatively charged VMSF for detection of clindamycin.

In this work, we demonstrate a flexible electrochemiluminescence sensor based on covering the electrode with vertically ordered mesoporous silica nanochannel films, which enable direct and sensitive detection of clindamycin in human serum. As shown in Figure 1, polyethylene terephthalate coated with indium tin oxide (PET-ITO), which is flexible, cheap, and suitable for the fabrication of portable, integrated, or disposable sensors, is used as the supporting electrode to grow VMSFs using the electrochemically assisted self-assembly (EASA) method. The significant enrichment toward the commonly used cationic ECL luminophores tris(2,2-bipyridyl) dichlororuthenium (II) [Ru (bpy)_3_^2+^] is proved resulting from the negatively charged VMSF nanochannels. As clindamycin can act as coreactant to promote the ECL of Ru (bpy)_3_^2+^, the developed VMSF/PET-ITO sensor can detect clindamycin with high sensitivity. In addition, high signal stability during the continuous blending process is revealed, showing the unique properties of flexible electrodes. Owing to the high antifouling characteristic of the VMSF, direct analysis of clindamycin in real biological sample, human serum, is also realized.

## Materials and Methods

### Chemicals and Materials

Cetyltrimethylammonium bromide (CTAB), tetraethoxysilane (TEOS), clindamycin hydrochloride, tris(2, 2′-bipyridyl)ruthenium (II), acetone, Na_2_HPO_4_, NaH_2_PO_4_, potassium hydrogen phthalate (KHP), NH_4_OH (25%), and H_2_O_2_ (30%) were purchased from Aladdin Chemistry Co., Ltd. (China). Polyethylene terephthalate coated with indium tin oxide (PET-ITO) with surface resistivity of 5.5–6.1 Ω/sq was purchased from Zhuhai Kaivo Optoelectronic Technology (China). Human blood serum was provided by the Center for Disease Control and Prevention (Hangzhou, China). Ultrapure water (18.2 MΩ cm) prepared using Mill-Q system (Millipore, United States) was used to prepare the aqueous solutions in this work.

### Measurements and Instrumentations

Transmission electron microscopic (TEM) images were taken on a JEM-2100 transmission electron microscope (JEOL Ltd., Japan) using an operating voltage of 200 kV. Before measurement, the VMSF was scrapped from PET-ITO and dispersed in ethanol. Copper grid was applied to support the VMSF in TEM characterization. Scanning electron microscopic (SEM) image was taken under a field-emission scanning electron microscopy (S-4800, Hitachi, Japan). CHI 832C electrochemical workstation (CH Instrument, China) and MPI-E workstation (Remex Analysis, China) were used for ECL measurement. The detection cell was fixed in a dark detection chamber and had a volume of ∼7 ml. Electrochemical measurements including cyclic voltammetry (CV) and electrochemical impedance spectroscopy (EIS) were carried out on an AutoLab electrochemical station (PGSTAT302N, Metrohm, Switzerland). Three electrode system was used in both electrochemical and ECL investigations. Briefly, bare or modified PET-ITO electrodes act as a working electrode. Ag/AgCl electrode (saturated with KCl solution) is applied as the reference electrode and Pt sheet electrode is the counter electrode.

### Growth of Vertically Ordered Mesoporous Silica Nanochannel Film on Polyethylene Terephthalate Coated With Indium Tin Oxide

To improve the hydrophilicity of PET-ITO, the electrode was immersed in a mixed solution containing ultrapure water/hydrogen peroxide/ammonium hydroxide (5:1:1, v/v) under dark conditions for 1.5 h. Then, the electrode was washed thoroughly with ultrapure water. The VMSF was equipped on PET-ITO using the electrochemically assisted self-assembly method. Briefly, 20 ml NaNO_3_ (0.1 mol/L, pH = 3), 20 ml ethanol, 2.833 g TEOS, and 1.585 g CTAB were mixed, and the obtained mixture was stirred for 2.5 h to obtain the precursor solution. For VMSF growth, a constant current (−0.35 mA/cm^2^) was applied on PET-ITO for 10 s. Then the electrode was quickly rinsed with ultrapure water. After drying under N_2_ stream, the electrode with surfactant micelles (SM) in the nanochannels (SM@VMSF/PET-ITO) was aged at 120°C for 10 h. Then, SM was removed by treating the SM@VMSF/PET-ITO in acetone and stirring for 30 min. Finally, the VMSF/PET-ITO with open nanochannels was obtained.

### Electrochemiluminescence Determination of Clindamycin

Phosphate-buffered saline (PBS, 0.01 mol/L, pH 7.0) containing Ru (bpy)_3_^2+^ (10 μmol/L) was used as the supporting solution to detect clindamycin. When different concentrations of clindamycin were added, the ECL signal on the VMSF/PET-ITO electrode generated during CV scan (potential ranged from 0 to 1.4 V at a scan rate of 100 mV/s) was recorded. The standard addition method was used to evaluate the reliability of the developed ECL sensor in analysis of a real sample. Briefly, the human serum was diluted by a factor of 50 with PBS. Then, ECL detection was carried out after adding a certain amount of clindamycin.

## Results and Discussion

### Facile Coupling Vertically Ordered Mesoporous Silica Nanochannel Film on Flexible Polyethylene Terephthalate Coated With Indium Tin Oxide Electrode and Charge Selectivity of Nanochannels

**FIGURE 2:**
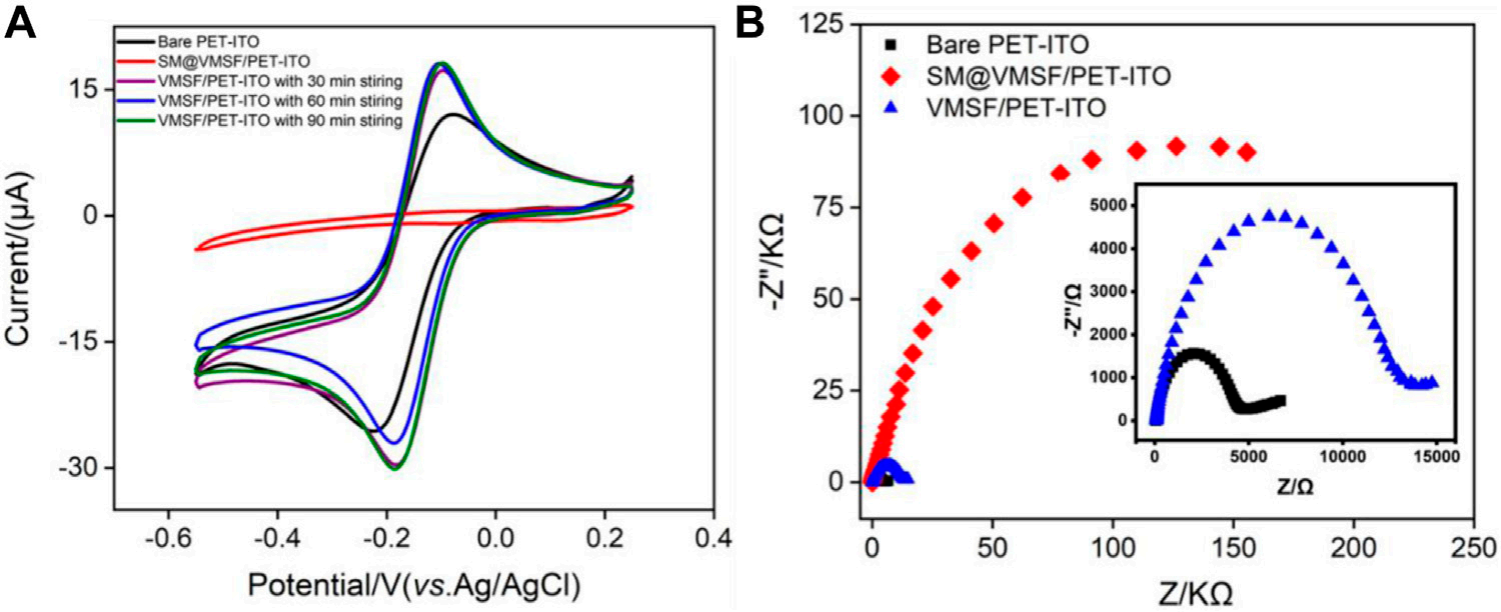
**(A)** Cyclic voltammetry (CV) curves on bare PET-ITO, VMSF/PET-ITO, or SM@VMSF/PET-ITO electrodes in Ru (NH_3_)_6_^3+^ solution (0.5 mmol/L in 0.05 mol/L KHP). The VMSF/PET-ITO electrode was prepared through the removal of SM using different soaking time with stirring in acetone as indicated. **(B)** EIS curves obtained on are PET-ITO, VMSF/PET-ITO, or SM@VMSF/PET-ITO in [Fe(CN)_6_]^3−/4^ (2.5 mmol/L in 1 mol/L KCl). Inset in **(B)** is the magnified plots of bare PET-ITO and VMSF/PET-ITO.

The flexible electrode is a research hotspot in recent years (Zhou et al., 2020b; İnce and Sezgintürk, 2021; Avelino et al., 2021). Compared with traditional hard electrodes, the flexible electrode has great potential in flexible or wearable sensors owing to the unique abilities of folding, bending, and multi-layer winding. Therefore, a simple and efficient modification of the flexible electrode to improve the detection sensitivity and antifouling ability in the complex sample is of great significance. Figure 1 is the schematic illustration for coupling VMSF on the flexible PET-ITO electrode using the electrochemically assisted self-assembly (EASA) method. Among the developed strategies to prepare the VMSF, the EASA method is fast and efficient which can achieve rapid growth of the VMSF within 5–30 s at room temperature. Briefly, the VMSF with a pore diameter of 2–3 nm is formed by using cetyltrimethylammonium bromide (CTAB) micelles (SM) as the template. The mechanism lies in the controlled condensation kinetics of siloxane around the SM template under the *in situ* pH gradient resulting from the reduction of protons and water molecules when a negative voltage is applied to the electrode. Researchers have systematically investigated the effects of experimental conditions on the film formation and properties of VMSF (Herzog et al., 2013). It has been proven that the electrode surface cannot be completely covered by the VMSF, if a short electrodeposition time and a small current density are employed. On the contrary, a long electrodeposition time and a large current density lead to a thick film, which in turn weakens the permeability of the film. If the concentration of silane precursor is too small, the electrodes will not be fully covered by the film. However, a higher precursor concentration will result in a film that is too thick and prone to cracking during the aging stage. After VMSF growth, the resulting electrode contains surfactant SM (SM@VMSF/PET-ITO), which blocks the nanochannels. The efficient removal of SM in the VMSF is the key to obtain open nanochannels. The most common method to remove SM is to soak the SM@VMSF/PET-ITO electrode in HCl-ethanol solution. However, PET will get damaged in HCl medium because of its hydrolysis reaction in strong acid. Thus, acetone is applied to remove SM in this work. Figure 2A shows the CV curves of Ru (NH_3_)^3+^ obtained on different electrodes including bare PET-ITO, SM@VMSF/PET-ITO and VMSF/PET-ITO, respectively. To investigate the effect of soaking time in acetone on the removal of SM, VMSF/PET-ITO electrodes prepared using different soaking time under stirring are studied. As shown, no Faraday current is observed on the SM@VMSF/PET-ITO electrode. This is because the micelles block the nanochannels so that the redox probes cannot reach the electrode surface. The result also proves that the VMSF is a complete film without cracks. In contrast, the VMSF/PET-ITO electrode without SM has significant current responses. In addition, the signal of Ru (NH_3_)_6_^3+^ on the VMSF/PET-ITO electrode remains unchanged after the VMSF/PET-ITO electrode is soaked in acetone for 30 min, indicating efficient removal of SM. Thus, 30 min of soaking in acetone is chosen for further investigation.

Compared with bare PET-ITO, the VMSF/PET-ITO electrode shows enhanced signal for Ru (NH_3_)^3+^, proving remarkable enrichment effect toward positively charges species. The deprotonation of silanols (pKa ∼2) on the surface of VMSF nanochannels leads to negatively charged surface, which attracts the positively charged Ru (NH_3_)^3+^ through electrostatic interactions. The successful growth of the VMSF and the charge selectivity of nanochannels are also confirmed by electrochemical impedance spectroscopy (EIS) obtained in the anionic [Fe(CN)_6_]^3-/4^ probe (Figure 2B). As shown, bare PET-ITO, SM@VMSF/PET-ITO, and VMSF/PET-ITO exhibit significantly different charge transfer resistance (*Rct*), which is related to the diameter of the curve semicircle in the high-frequency region. When SM exist in nanochannels, the SM@VMSF/PET-ITO electrode exhibits the largest *Rct* owing to the blocking of nanochannels. When SM is removed, the *Rct* for VMSF/PET-ITO electrode remarkably decreases. However, the *Rct* for the VMSF/PET-ITO electrode is still larger than that of bare PET-ITO, indicating the electrostatic repulsion of negatively charged nanochannels to negatively charged probes.

### Morphology of Vertically Ordered Mesoporous Silica Nanochannel Film Nanochannels

**FIGURE 3:**
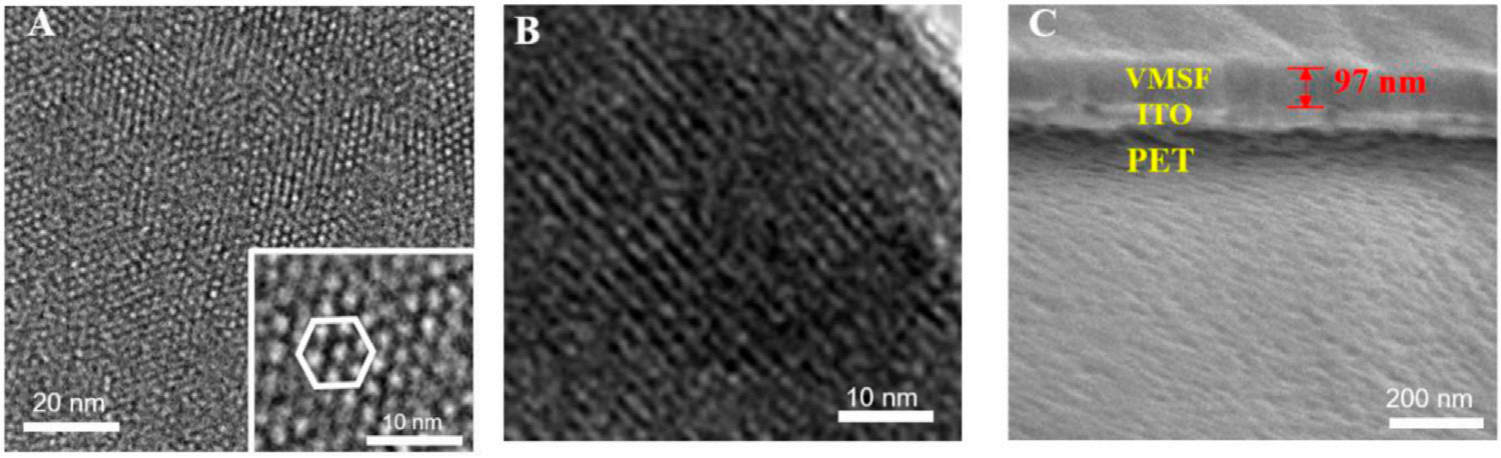
**(A)** Top-view and **(B)** cross-sectional TEM images of VMSF. Inset in **(A)** is the image at high magnification. **(C)** Cross-sectional SEM images of VMSF/PET-ITO electrode.

The structure and morphology of the VMSF are characterized by TEM and SEM. Figure 3A is the top-view TEM images of the VMSF at different magnification. As shown, the VMSF has a uniformly distributed nanopore structure without cracks or defects in the observed range. The highly ordered nanopores have a diameter of 2–3 nm. The cross-sectional TEM image of the VMSF in Figure 3A reveals nanochannels parallel to each other. The cross-sectional SEM image of the VMSF/PET-ITO electrode shows a three-layered structure, which corresponds to PET substrate, ITO layer, and VMSF layer from bottom to top. In addition, VMSF has a flat structure with a thickness of ∼97 nm.

### Enhanced Electrochemiluminescence by Clindamycin and Vertically Ordered Mesoporous Silica Nanochannel Film Nanochannels

**FIGURE 4:**
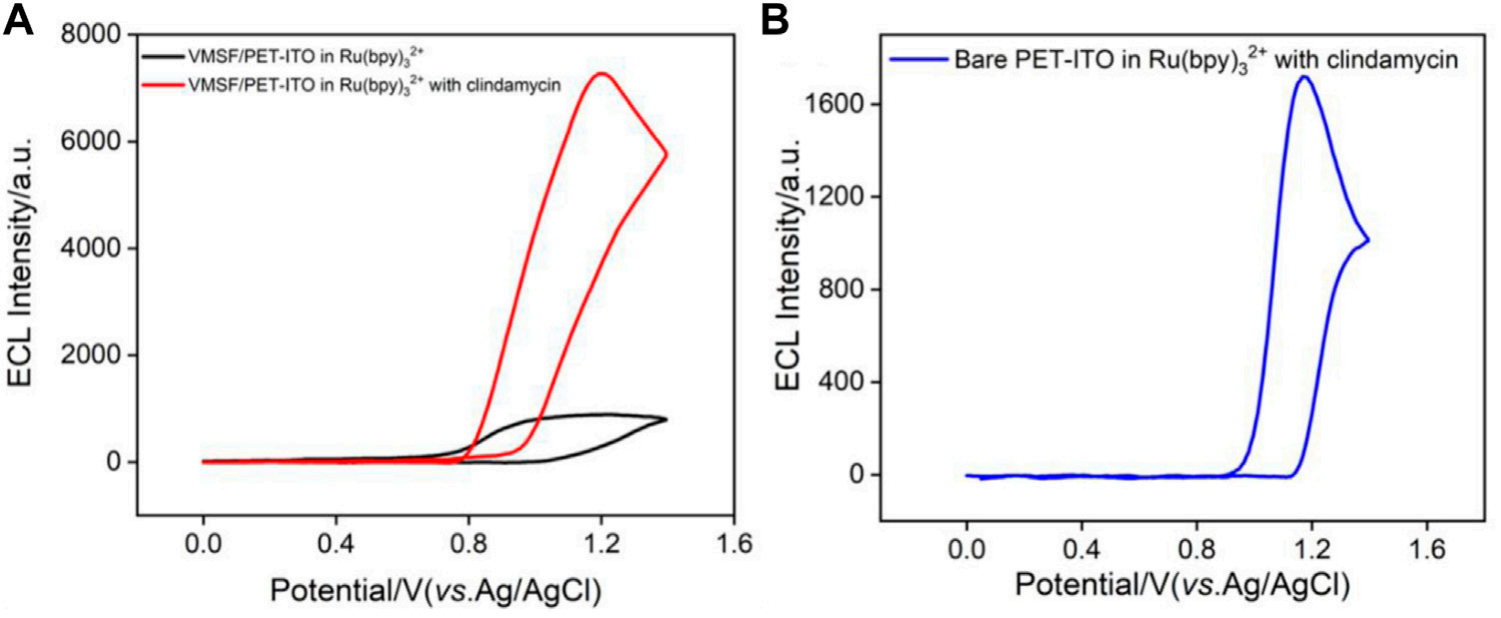
**(A)** ECL signals obtained on VMSF/PET-ITO electrode in PBS containing Ru (bpy)_3_^2+^ (10 μmol/L) before and after adding clindamycin (30 μmol/L). **(B)** ECL signal obtained on bare PET-ITO in the mixture of Ru (bpy)_3_^2+^ (10 μmol/L) and clindamycin (30 μmol/L).

Electrochemiluminescence has the unique advantage of simple instruments, low background, and easy operation. Although many ECL luminophores such as acridan ester, ruthenium chelate, luminol, and nanoemitters have been developed, tris(2,2′-bipyridyl) ruthenium (II) [Ru (bpy)_3_^2+^] is still widely used due to high stability, solubility, and reversible electrochemical behavior (Zhou et al., 2019a; Zhou et al., 2020a; Ding et al., 2020; Li et al., 2020). As shown in Figure 4A, when clindamycin is added to Ru (bpy)_3_^2+^, the ECL signal significantly enhances, indicating that clindamycin can promote the ECL emission. Owing to the quaternary amine structure in the clindamycin molecule, the possible ECL mechanism might be similar to that of Ru (bpy)_3_^2+^/tri-n-propylamine (TPrA) system. The ECL reaction pathway can be elucidated in the following equations (Eqs 1–5). Briefly, Ru (bpy)_3_^2+^ can be oxidized to Ru (bpy_)3_^3+^ when an oxidation potential of 1.4 V is applied to the electrode. At the same time, clindamycin is oxidized to radical cation (clindamycin^**.**+^) followed with deprotonation to form the reduced product 1(clindamycin^**.**^ **)**, which undergoes redox reaction with Ru (bpy)_3_^3+^ to generate the excited state [Ru (bpy)_3_^2+*^]. Then, ECL is observed when the excited state returns to the ground state.

$$\text{Ru}{\left(\text{bpy}\right)}_{3}^{2+}-\;{\text{e}}^{-}\to \text{Ru}{\left(\text{bpy}\right)}_{3}^{3+}$$ (1)

$$\text{Clindamycin}\;-\;{\text{e}}^{-}\to \text{Clindamyci}{\text{n}}^{•+}$$ (2)

$$\text{Clindamyci}{\text{n}}^{•+}\to \text{Clindamyci}{\text{n}}^{•}+\;{\text{H}}^{+}$$ (3)

$$\text{Ru}{\left(\text{bpy}\right)}_{3}^{3+}+\;\text{Clindamyci}{\text{n}}^{•}\to \text{Ru}{\left(\text{bpy}\right)}_{3}^{2+*}+\;\text{Clindamycin}\;\text{fragment}$$ (4)

$$\text{Ru}{\left(\text{bpy}\right)}_{3}^{2+*}\to \text{Ru}{\left(\text{bpy}\right)}_{3}^{2+}+\;\text{hv}$$ (5)

It is worth noting that the ECL signal on the VMSF/PET-ITO electrode is about 7 times of that on the bare PET-ITO electrode (Figure 4B), indicating that the nanochannels of the VMSF have significant signal amplification effect. This was attributed to the enrichment of positively charged ECL probes by VMSF nanochannels.

### Optimized Conditions for Electrochemiluminescence Detection of Clindamycin

To achieve the best detection performance, the concentration and pH of the supporting electrolyte are optimized. As shown in Supplementary Figure S1A (in supporting information, SI), the ECL intensity gradually decreases when the ionic strength of the supporting buffer increases. This is because the thickness of the electric double layer of VMSF nanochannels is inversely proportional to the ionic strength. The increase in thickness of the electric double layer of the nanochannels enhances the electrostatic adsorption of Ru (bpy)_3_^2+^, resulting in more ECL probes on the electrode surface. As can be seen from Supplementary Figure S1B (SI), the ECL intensity on the electrode gradually increases as the pH of the buffer increases from 4 to 8. This might be ascribed to the enhanced deprotonation of the radical cation of clindamycin (clindamycin^**.**+^) to form strong reducing agent (clindamycin, Eq. 3), which facilitates the redox reaction with Ru (bpy)_3_^3+^ and the formation of the excited state (Ru (bpy)_3_^2+*^) (Leland and Powell, 1990). The phenomenon further confirms the coreactant mechanism for the enhanced ECL signal. The decrease of ECL signal at pH 9 might result from the instability of the VMSF at high alkaline medium. Considering the stability of the VMSF and the neutral environment of common physiological samples, pH 7 is chosen for further experiments.

### Sensitive Electrochemiluminescence Detection of Clindamycin

**FIGURE 5:**
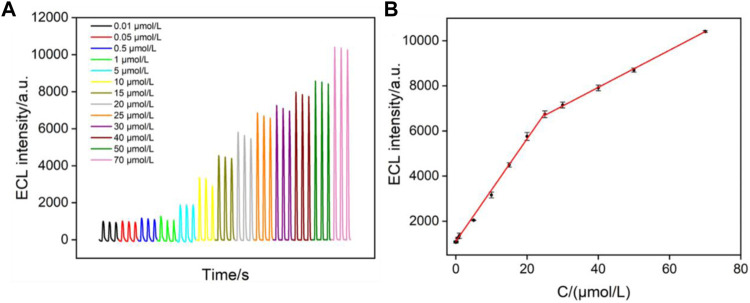
**(A)** ECL intensity of Ru (bpy)_3_^2+^ in the presence of different concentrations of clindamycin. **(B)** Linear dependence between the ECL intensity and the concentration of clindamycin.

Under the optimized conditions, ECL detection of clindamycin is investigated. Figure 5A illustrates the ECL intensity on the VMSF/PET-ITO electrode in the presence of different concentrations of clindamycin. The ECL intensity (I_ECL_) is linearly correlated to the concentration of clindamycin (C) in the range from 10 nmol/L to 25 μmol/L (I_ECL_ = 227.2C + 1,106, *R* ^2^ = 0.9941) and from 25 μmol/L to 70 μmol/L (I_ECL_ = 82.45C + 4,635, *R* ^2^ = 0.9995) (Figure 5B). The limit of detection (LOD) is based on a signal-to-noise ratio of 3 (*S/N* = 3) is 4 nmol/L. Owing to the enrichment effect of VMSF nanochannels toward Ru (bpy)_3_^2+^, the LOD from the VMSF/PET-ITO electrode is lower than that obtained using square wave voltammetry (SWV) detection on a glassy carbon electrode modified with graphene oxide and gold nanoparticles within a film of cross-linked chitosan with epichlorohydrin (AuNP-GO-CTS-ECH/GCE electrode) or CV detection on edge-plane pyrolytic graphite electrode (EPPG) (Wong et al., 2016; Hadi and Honarmand, 2017). The LOD is also lower than that obtained using capillary electrophoresis (CE) or CE-ECL (Wang et al., 2008; Paul et al., 2017). Thus, the constructed ECL sensor has the advantages of simple preparation and high detection performance.

### High Stability of Vertically Ordered Mesoporous Silica Nanochannel Film/Polyethylene Terephthalate Coated With Indium Tin Oxide Electrode Under Continuous Bending

**FIGURE 6:**
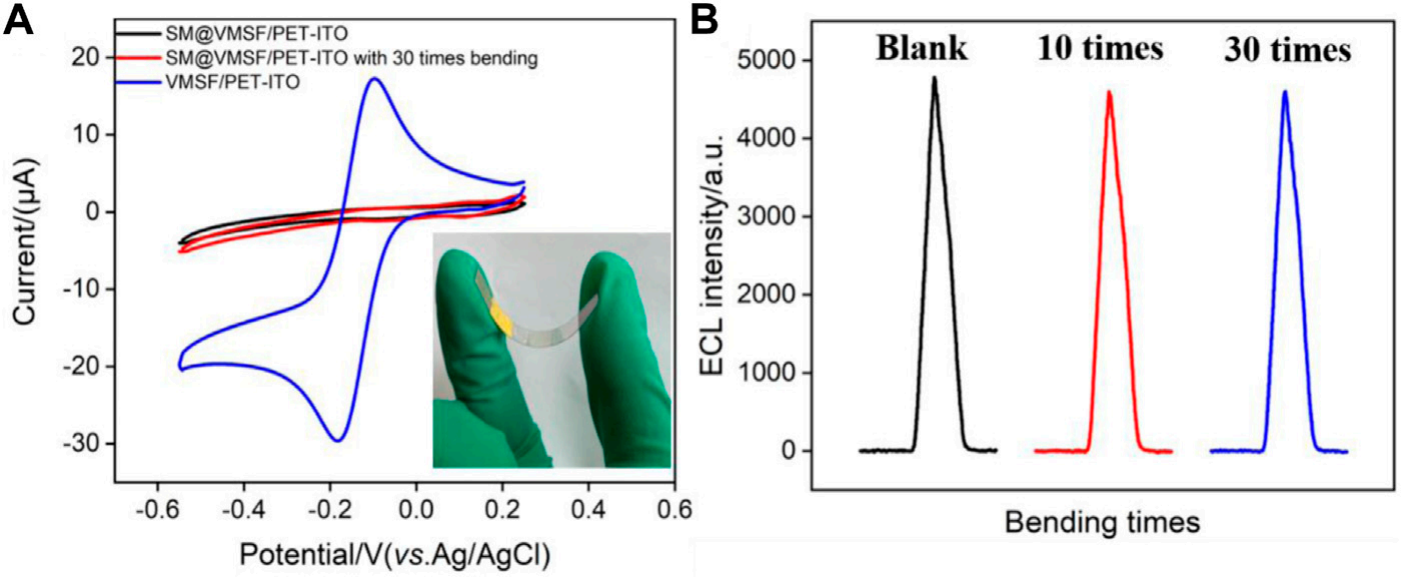
**(A)** CV curves of SM@VMSF/PET-ITO and VMSF/PET-ITO electrodes in Ru (NH_3_)_6_^3+^ solution (0.5 mmol/L in 0.05 mol/L KHP). Inset is the image of the electrode in case of bending. **(B)** ECL signals obtained on original or blended VMSF/PET-ITO in the mixture containing Ru (bpy)_3_^2+^ (10 μmol/L) and clindamycin (15 μmol/L) in PBS solution.

The signal stability under large deformation is a key factor in evaluating the performance of flexible electrodes. We compared the signals on the VMSF/PET-ITO electrode before and after continuous bending. The micelle-containing electrode, SM@VMSF/PET-ITO electrode, is first investigated with continuous bending for 30 times. As shown in Figure 6A, CV curves of Ru (NH_3_)_6_^3+^ on the original or bended electrode are quite similar. There was no significant Faraday signal before and after folding of the electrode. Thus, no crack of the VMSF layer appears during the continuous bending process, proving high stability of the flexible electrode. When the micelles are removed from the electrode that was folded 30 times, the opening of the nanochannels leads to significant electrochemical signals of the redox probe. In addition, the stability of the ECL signal on the electrode during the continuous bending is also investigated. As shown in Figure 6B, the electrode before and after bending for 10 or 30 times exhibits unchanged ECL intensity, indicating high signal stability. This phenomenon is attributed to the nanoscale thickness of the VMSF and the high coupling stability with the electrode substrate.

### Antifouling of Vertically Ordered Mesoporous Silica Nanochannel Film/Polyethylene Terephthalate Coated With Indium Tin Oxide Electrode and Real Sample Analysis

**FIGURE 7:**
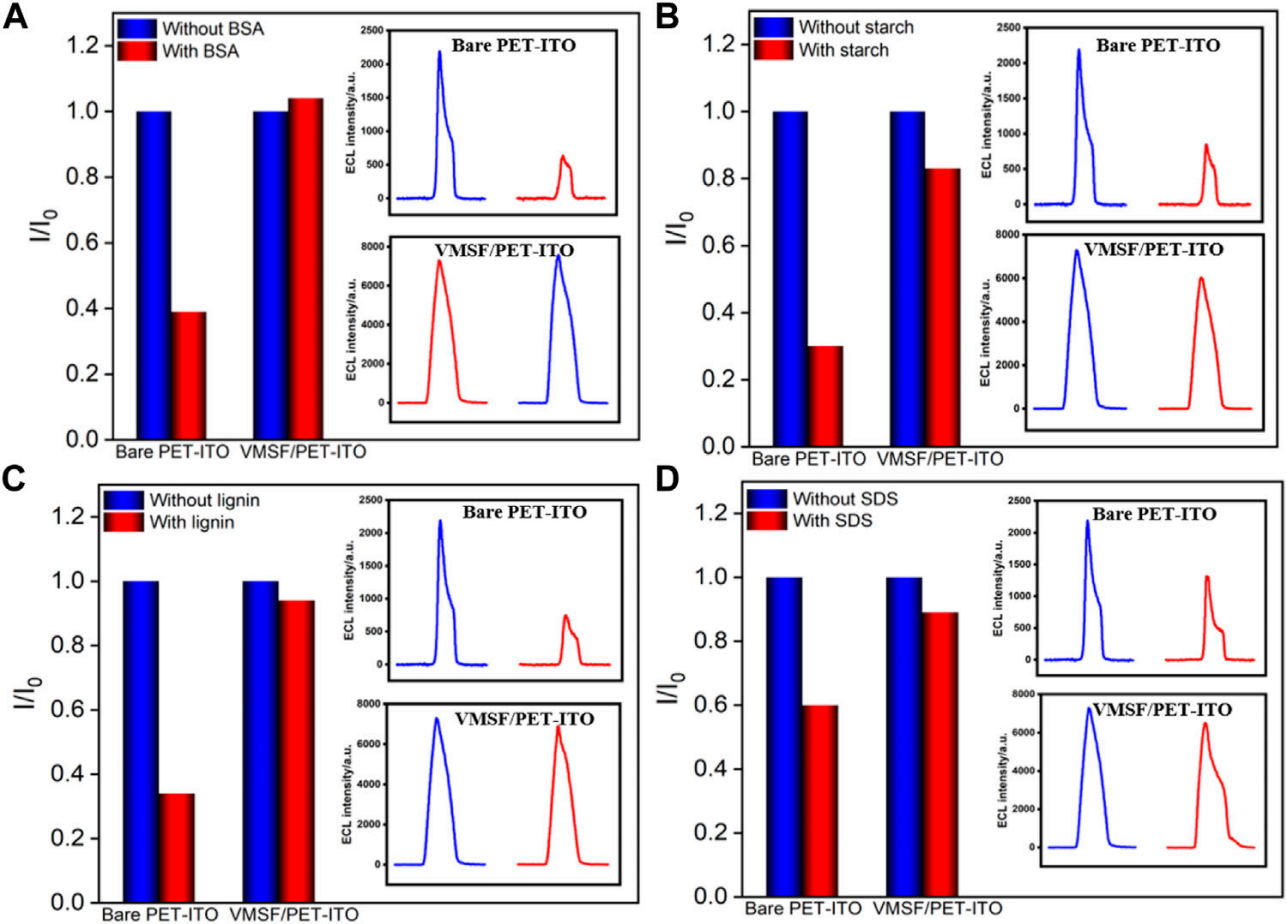
Normalized ECL intensity ratio on bare PET-ITO or VMSF/PET-ITO toward clindamycin (30 μM). I and I_0_ represent the ECL intensity obtained in the absence and present of 50 μg/ml **(A)** BSA, **(B)** starch, **(C)** lignin, or **(D)** SDS in PBS (0.01 mol/L, pH = 7). The insets are the corresponding ECL intensity obtained on bare PET-ITO and VMSF/PET-ITO in the absence or presence of fouling species.

Biological, food, and environmental samples usually have complex matrix containing proteins, starch, surfactants, etc., which often contaminates the electrode surface and reduces signal accuracy. Thus, improving the antifouling ability of the electrode is of great significance for direct electroanalysis of complex samples. To evaluate the antifouling ability of the developed VMSF/PET-ITO sensor, bovine serum albumin (BSA), starch, lignin and sodium dodecyl sulfonate (SDS) are applied as the possible interfering substances. The ECL of Ru (bpy)_3_^2+^ with clindamycin in the absence or presence of one of these substances on both VMSF/PET-ITO and PET-ITO electrodes are given in Figure 7. As shown, the ECL signal significantly reduced on PET-ITO when one of the aforementioned substances is added, indicating remarkable fouling of the electrode. On the contrary, no significant change of ECL signal is observed on the VMSF/PET-ITO electrode, indicating high antifouling performance resulting from the remarkable molecular sieving ability of the VMSF.

Considering the antifouling ability of the VMSF, the developed VMSF/PET-ITO sensor is applied to detect clindamycin in human serum samples (diluted by a factor by 50) using the standard addition method (Supplementary Table S1 in SI). The recovery rate of other three artificial added clindamycin concentration is between 97.6 and 104.0% with low relative standard deviation (RSD, ≤4.7%), indicating good reliability in the direct measurement of clindamycin in real complex samples.

## Conclusion

In summary, we demonstrate a cost-effective and disposable electrochemiluminescence sensor based on the flexible electrode covered with a vertically ordered mesoporous silica nanochannel film for sensitive detection of clindamycin. The polyethylene terephthalate coated with indium tin oxide (PET-ITO) is applied as the flexible electrode to easily grow the VMSF using the electrochemically assisted self-assembly (EASA) method. The negatively charged VMSF nanochannels exhibit significant enrichment toward the commonly used cationic ECL luminophores, tris(2,2-bipyridyl) dichlororuthenium (II) [Ru (bpy)_3_^2+^]. Using the enhanced ECL of Ru (bpy)_3_^2+^ by clindamycin, the developed VMSF/PET-ITO sensor can detect clindamycin with high sensitivity. Owing to the nanoscale thickness of the VMSF and the high coupling stability with the electrode substrate, the developed VMSF/PET-ITO sensor exhibits high signal stability during the continuous bending process. Considering high antifouling characteristic of the VMSF, direct analysis of clindamycin in human serum is realized. Because the PET-ITO electrode is easy to be miniaturized or patterned, the proposed strategy of modifying flexible electrode based on the nanochannel is expected to open up a new research field in flexible, wearable, or miniaturized sensors.

## Data Availability

The original contributions presented in the study are included in the article/Supplementary Material; further inquiries can be directed to the corresponding author.
