# Supplementary material for: A Flexible Electrochemiluminescence Sensor Equipped With Vertically Ordered Mesoporous Silica Nanochannel Film for Sensitive Detection of Clindamycin
Source: Front Chem. 2022 Apr 6;10:872582. doi: 10.3389/fchem.2022.872582 (PMC9019221; doi:10.3389/fchem.2022.872582)
Supplement: Supplementary file 1 [file DataSheet1.docx]

**Figures**


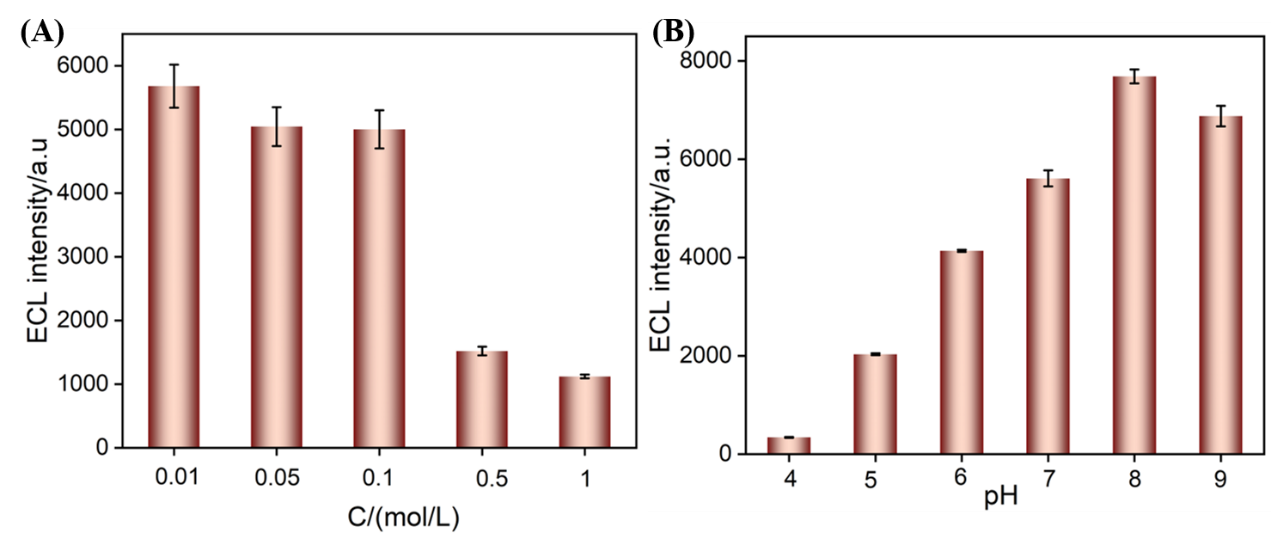


Figure S1. ECL signals obtained on VMSF/PET-ITO electrode in the mixture containing Ru(bpy)_3_^2+^ (10 μmol/L) and clindamycin (30 μmol/L) at (A) different concentration (pH= 7) or (B) pH (0.01 mol/L) of PBS.

**Tables**

Table S1 Determination of clindamycin in real sample.

| Sample | Added(μM) | Total found (μM) | Recovery (%) | RSD (%) |
| --- | --- | --- | --- | --- |
| Serum^a^ | 3.00 | 3.12 | 104.0 | 4.7 |
|  | 10.0 | 9.76 | 97.6 | 2.8 |
|  | 20.0 | 20.1 | 100.5 | 3.3 |
|  | 25.0 | 25.1 | 100.4 | 2.2 |

^a^: diluted with PBS (0.01 M, pH=7) for 50 times.
